# Radiotherapy for Primary Urethral Carcinoma (PUC): An Illustrative and Narrative Review

**DOI:** 10.1016/j.ctro.2025.101074

**Published:** 2025-11-10

**Authors:** Shreya Dhingra, Arunima Nagar, Amandeep Arora, Maneesh Singh, Priyamvada Maitre, Ankit Misra, Mahendra Pal, Amit Joshi, Santosh Menon, Herney Andres Garcia-Perdomo, Philippe Spiess, Gagan Prakash, Vedang Murthy

**Affiliations:** a Department of Radiation Oncology, Tata Memorial Centre, Homi Bhabha National Institute, Mumbai, India; b Department of Surgical Oncology, Tata Memorial Centre, Homi Bhabha National Institute, Mumbai, India; c Department of Medical Oncology, Tata Memorial Centre, Homi Bhabha National Institute, Mumbai, India; d Department of Pathology, Tata Memorial Centre, Homi Bhabha National Institute, Mumbai, India; e Division of Genito-urinary Oncology Department of Surgery School of Medicine, Universidad del Valle, Cali, Colombia; f Department of Genito-urinary Oncology, Moffitt Cancer Center, Florida, United States

**Keywords:** Urethral carcinoma, Organ preservation, Radiotherapy, Brachytherapy, Review

## Abstract

Primary urethral carcinoma (PUC) is a rare malignancy with a complex and site-specific management paradigm. While surgery remains the mainstay for many cases, advances in modern radiotherapy have facilitated organ preservation without compromising oncologic outcomes. This narrative review outlines the clinicopathological features, diagnostic evaluation, and evolving role of radiotherapy in the management of PUC. An illustrative case of a young male with high-grade urothelial carcinoma of the bulbar urethra managed successfully with definitive external beam radiotherapy is presented. We explore the rationale, technique, and outcomes associated with radiotherapy, including external beam and brachytherapy modalities, across disease stages. For locally advanced cases, chemoradiotherapy offers an organ-sparing alternative to mutilating surgery, with promising control rates and acceptable toxicity. This article aims to collate current evidence, highlight gaps, and support the integration of personalised, multidisciplinary care in this rare disease context.

## Introduction

Primary urethral cancer (PUC) is a rare diagnosis accounting for less than 1 % of all genitourinary malignancies [1]. Owing to rarity of the tumour, there is lack of robust data for guiding management strategies. Historically, PUC management has mirrored the treatment workflow followed for penile cancer. This usually consists of multimodal treatment comprising functionally debilitating surgeries to incorporate wide margins followed by adjuvant treatment with radiation therapy (RT) and systemic therapy. However, more recently, modern radiotherapy has ushered a renewed emphasis on organ and function preservation for maintaining quality of life without compromising oncological outcomes. Here, we present an illustrative case of male urethral carcinoma treated with external beam radiotherapy (EBRT) and briefly review the existing literature on the management of PUC with special emphasis on the role of RT [2].

## Case summary

**Fig. 1:**
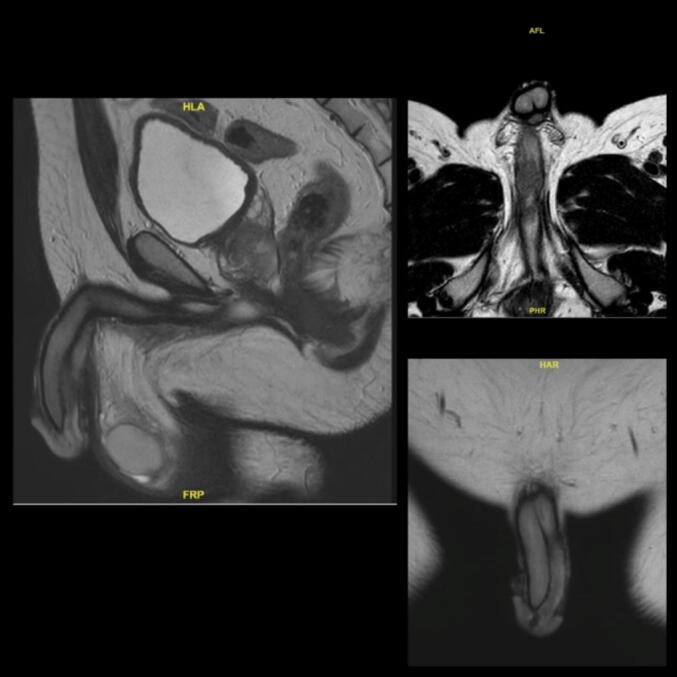
T2- weighted sequence of MRI Pelvis depicting the disease in bulbar urethra at baseline.

A 40-year-old fit man with a history of blood streaks in urine and semen with no accompanying lower urinary tract symptoms (LUTS) or blood dyscrasias, presented with acute urinary retention (AUR) requiring catheterisation. A broad-based pedunculated tender mass of 2x2cm, firm to hard consistency, was palpable in the mid bulbar-urethra, confirmed on cystoscopy, and a transurethral resection of the urethral tumour (TUR-UT) was performed. Histopathology was suggestive of high-grade urothelial carcinoma (HG-UC) with squamous differentiation and invasion of muscularis propria. MRI showed a residual 1.5 cm thick lesion along the inferior aspect of the distal bulbar and penile urethra involving the corpus spongiosum for a length of about 6.2 cm (Fig. 1). Regional and distant metastatic spread were ruled out with a fluorodeoxyglucose-18 positron emission tomography (FDG-PETCT) imaging. The disease was staged T2N0M0, per the AJCC TNM classification (8th edition) [3] and the patient was given the options of RT or urethrectomy with total penectomy after a multi-disciplinary joint clinic meeting. After shared decision- making with his wife and family, the patient opted for organ preservation with RT.

**Fig. 2:**
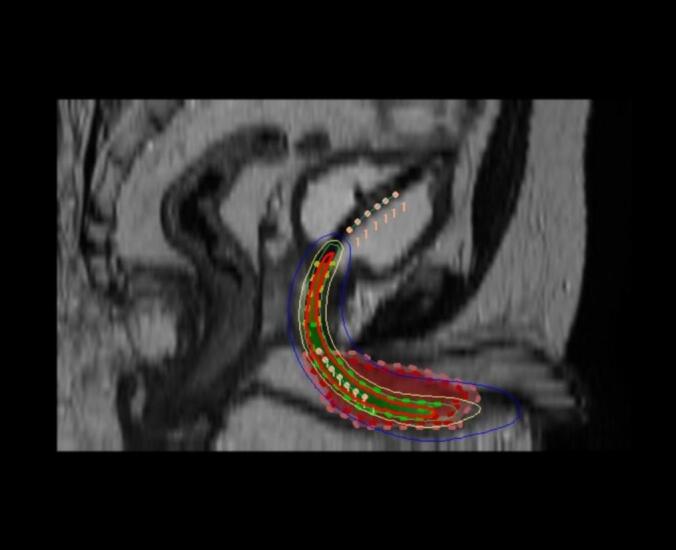
HDR Intraluminal Brachytherapy plan- sagittal view: Green dotted line: Urethra, Red dotted line: Gross tumour volume, Pink dotted line: Clinical target volume, pink dots: Source dwell positions, Red continuous line: 200 % isodose curve, Green continuous line: 150 % isodose curve, Yellow continuous line: 100 % isodose curve, Blue continuous line: 50 % isodose curve. The gross tumour volume was inadequately covered with 100 % isodose curve line. (For interpretation of the references to colour in this figure legend, the reader is referred to the web version of this article.)

The initial plan was to proceed with high-dose-rate (HDR) intraluminal brachytherapy using transurethral route. A Foley’s catheter was inserted to facilitate intraluminal source placement, and a treatment plan was generated on the planning CT. However, given the circumferential tumour thickness exceeding 15  mm, it was challenging to strike a balance between achieving adequate tumour coverage up to its peripheral margins and adhering to urethral dose constraints, making intraluminal brachytherapy planning difficult [4]. Consequently, the option of brachytherapy was not pursued. The brachytherapy plan has been depicted in Fig. 2.

The patient was planned for EBRT in supine position with knee support and was instructed to maintain a comfortably full bladder and empty rectum for reproducibility. Gross tumour volume (GTV) was delineated on the T2 weighted sequence of MRI fused with planning contrast enhanced CT. For clinical target volume (CTV), 5 mm isotropic margin was grown to cater to surrounding micro-metastases, and was extended cranially to include the posterior urethra till neck of bladder [5]. An isotropic margin of 5 mm was used to generate the planning target volume (PTV) based on institutional protocols. Volumetric modulated arc technique (VMAT) with 6MV photons was used for radiation therapy planning. A dose of 55 Gy was prescribed to the PTV, with a simultaneous integrated boost planned to deliver 60 Gy to the GTV, all over 20 once-daily fractions across 4 weeks, using daily image guidance for treatment delivery. The hypo-fractionated dose regimen was derived from treatment protocols used in bladder cancer [6].

**Fig. 3:**
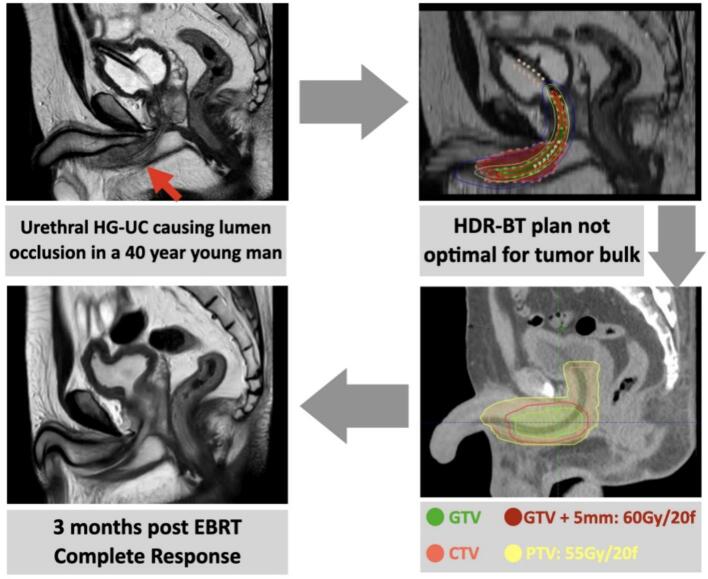
Illustrative summary of the case.

The mean doses received by the organs-at-risk were: anorectum- 12.2 Gy, bladder-13 Gy, and genitalia- 2.5 Gy, all of which were well within the recommended constraints for pelvic radiotherapy [7,8]. Patient tolerated the treatment well with no acute gastro-intestinal and mild (CTCAE grade 1) acute genitourinary toxicities. Clinical and radiological assessment with MRI, 3 months after radiation completion confirmed a complete response to treatment (Fig. 3). At 15 months post-RT, he developed urinary obstruction (stricture) refractory to serial dilatations and clean intermittent self-catheterisation (CISC) requiring urethroplasty. This was regarded as Late CTCAE Grade 3 urinary toxicity per the Common Terminology Criteria for Adverse Events (CTCAE) version 5.0. The patient has now completed 2.5 years of follow up and is clinically and radiologically disease free.

## Discussion and narrative review of PUC

In the presented case of a 40-year-old man with high-grade urothelial carcinoma of the bulbar urethra who opted for organ-preserving radiotherapy, external beam radiotherapy, while brachytherapy was not technically feasible, achieved a long-term control at the cost of late complications like urethral stricture requiring urethroplasty. This case highlights the challenges of treating HG-UC of the bulbar urethra, the shift from brachytherapy to EBRT for organ preservation and balancing adequate treatment response with limited regional toxicity. The importance of multidisciplinary care in defining optimal treatment strategies cannot be overemphasized. While achieving disease control, late toxicities like urethral stricture underscore the need for long-term monitoring for potential delayed recurrence or locoregional complications.

### Epidemiology, clinicopathological characteristics and outcomes of urethral cancer

PUC is a rare entity and most commonly, lesions identified in the urethra are metastatic deposits from primary tumours in the bladder [1].The etiopathogenesis differs for males and females. The incidence is almost three times more in males than in females [9]. The aetiology in males is attributable to chronic irritation caused by venereal diseases (mainly condylomata associated with Human Papilloma Virus) (24–37 %), long-standing stricture of the urethra and repeated catheterisation (35–54 %), trauma (7 %), and polyp transformation (2 %) along with predisposing factors like history of cancers of the urogenital tract and irradiation [10]. History of urethral diverticula and recurrent urinary tract infections predisposes to PUC in females [11]. The average age of presentation is >60 years, peaking in the eighth decade according to a recent Surveillance, Epidemiology, and End Results analysis [12,13]. However, 10 % of urethral tumours occur in ages younger than 40 years [10], as in our patient. No genetic or geographic predisposing factors have been validated in previous studies. The predominant histology in males is urothelial carcinoma (UC) followed by Squamous cell carcinoma (SCC) and Adenocarcinoma (AC) while the order seems to be reversed in females [13]. The natural history of distal urethral (penile and bulbar) disease is similar to that of penile cancer while the cancers originating in proximal urethra (prostatic and membranous) have histopathological similarities to bladder cancer and invade deeper structures first. Patients often present with locally advanced disease due to subtle initial symptoms and indolent course, with haematuria/haematospermia in most cases followed by extra-urethral mass, bladder outlet obstruction, pelvic pain and fistula or abscess [14]. The 5-year survival rate for PUC is 54 % [13]. Factors like older age, proximal urethral lesion and size of tumour, non-UC histology, limited extent of surgical resection, advanced stage, higher histological grade, and concomitant deposits in bladder or prostate, portend a poorer prognosis [1].

### Clinical evaluation

Evaluation of suspected PUC begins with a thorough physical examination including inspection of the external genitalia and a digital rectal exam in men, and pelvic exam with urethral palpation in women, along with inguinal node examination. Urine cytology has limited diagnostic value, detecting PUC in only 59 % of biopsy-confirmed cases [10]. Diagnostic work-up often involves examination under anaesthesia and cystoscopy, for tissue diagnosis, disease staging and detection of any skip lesions in the urinary tract. The imaging modality of choice for assessing the local extent of PUC is a T2-weighted MRI of the pelvis. For systemic staging, FDG-PETCT is recommended per EAU guidelines, as urethral tumours primarily spread via direct extension to adjacent structures but also carry a 15 % risk of distant metastases [9]. Staging of the disease is done as per AJCC TNM classification (8th edition).

### Management of urethral cancer

The management of PUC depends on disease location, size, stage and patient factors. Location and gender are the two most vital determinants of the choice of modality [15]. Early-stage tumours (Tis-2N0 M0) are treated with single-modality therapy, either surgery (partial/total urethrectomy) or definitive RT. Locally advanced disease requires a multimodal approach, combining surgery, radiation, and chemotherapy. Metastatic or recurrent cases are managed with systemic chemotherapy, immunotherapy, and palliative interventions for symptom control. Table 1 summarises the treatment options in different stages of PUC.

**Table 1:** Structured overview of treatment options for primary urethral cancer in males and females.

Tumor location	Gender	Stage	Treatment options
Distal PUC	Females	Tis, Ta	Open excision, electro-resection, fulguration, neodymium-doped yttrium aluminum garnet (Nd:YAG) laser or CO2 laser vaporization-coagulation
		T1	Brachytherapy or combination of brachytherapy and EBRT
		T2	Brachytherapy or combination of brachytherapy and EBRT
		T3 or recurrent	Anterior exenteration with urinary diversion
	Males	Tis, Ta	Resection and fulguration
		T1, T2	Segmental urethral excision, preserving penile corpora
		T3	Penile amputation
		Any stage (if surgery refused)	Radiation therapy, combination chemotherapy and radiation
Proximal PUC	Females	Any stage	Exenteration surgery with urinary diversion, pelvic lymphadenectomy
		Tumors ≤ 2 cm	Radiation alone, non-exenteration surgery, or combination therapy
	Males	Any stage	Radical cysto-prostatectomy with en bloc penectomy, pelvic lymphadenectomy
		Any stage (preoperative approach)	Radiation or combination chemotherapy and radiation, followed by surgery
Any	Any	T3-4 N0-2	Neoadjuvant chemotherapy and surgery, Definitive chemoradiotherapy, Surgery followed by adjuvant CTRT

## Treatment for localized disease (early-stage tumours)

Single-modality treatment is preferred over multimodality for early-stage PUCs (Tis-2 N0 M0). Tumours of the distal urethra have a more favourable prognosis compared to those arising in the proximal urethra [1]. Consequently, efforts to enhance functional outcomes are particularly relevant in this subset of patients.

### Surgery

Surgery is often the first choice for early-stage PUCs, but it comes with some disadvantages and challenges of survivorship. Radical cysto-prostatectomy with en bloc penectomy is the standard approach for localized tumours in the proximal urethra in males. Penile-sparing surgery is recommended for distal urethral tumours to preserve urinary and sexual function, involving tumour excision with a margin of 2 cm healthy tissue. Postoperative complications, including infection, bleeding, and stricture formation, occur in about a quarter of cases. Additionally, over half the patients experience erectile dysfunction, altered body image perception, and a decline in quality of life [16,17,18]. Radical urethrectomy with peri-urethral tissue removal extending to the bulbocavernosus muscle bilaterally and distally, including adjacent soft tissue up to the pubic symphysis and bladder neck, offers the best chance of local cure in distal PUC. The outcomes in proximal tumours are dismal due to their higher likelihood of invasion and nodal spread, even with exenteration surgery and urinary diversion, reported 5-year survival rates lie between 10 to 20 % [19]. Postoperative incontinence has been reported in up to 42 % of patients [19]. For proximal tumours in both males and females, pelvic lymphadenectomy is routinely performed. In both proximal and distal tumours, only if inguinal lymph nodes are palpable, ipsilateral lymph node dissection is indicated following frozen section confirmation of malignancy [10]. Dynamic sentinel node biopsy is also emerging as a novel approach [20]. For locally advanced carcinomas, primary surgical resection alone offers a limited prognosis, with a 5-year overall survival rate of approximately 20 %–30 % [5]. Ablative techniques (Transurethral resection (TUR), electro-resection, fulguration, or laser vaporization-coagulation) can be offered for distal Tis, Ta, T1 lesions but are oncologically inadequate, associated with a 16 % local failure rate and a 50 % cancer-specific survival (CSS) rate [21].

### Radiation therapy (RT)

#### Definitive external beam RT

Radiation therapy, including EBRT or brachytherapy, could offer a vital alternative for functional organ preservation and its importance in the management of PUC has become well established over time. A retrospective study involving 84 patients who underwent radiotherapy as the sole treatment modality reported local control rates of 72 %, 65 %, and 64 % at 1, 2, and 5 years, respectively. The majority of recurrences occurred within the first two years, with patients exhibiting full-length urethral involvement demonstrating significantly poorer local disease control. The disease-free survival rates at 5, 10, and 15 years were 49 %, 45 %, and 42 %, respectively [22]. Use of definitive external beam RT in patients unfit for surgery or who prefer organ preservation is currently recommended [23,24]. In addition, for tumours of the proximal urethra with size ≤ 2 cm, radiation alone or a combination of RT with non-exenteration surgery, can provide excellent outcomes. In tumours with larger size, preoperative RT with or without chemotherapy may be utilized to reduce tumour burden, improve resectability, and decrease recurrence rates. The NCCN guidelines recommend conventionally fractionated doses of 66–70 Gy delivered to gross disease with a margin to encompass the microscopic spread in urothelial carcinomas and SCC, using conformal techniques [25]. Coverage of regional nodal basins (inguinal and low pelvic nodes for females and distal male tumours while pelvic nodes for proximal male tumours) is strongly recommended [25]. Earlier studies have reported that radiotherapy was generally well tolerated, with serious adverse effects occurring in approximately 15–20 % of cases [19]. However, these findings are based from the era of conventional radiotherapy. With advancements in conformal radiation techniques and the integration of brachytherapy, the incidence of severe toxicities has significantly decreased

#### Brachytherapy

Brachytherapy has shown excellent outcomes in definitive settings for early stages, achieving 100 % local control, as reported by Merten et al. [26]. For T1-2 tumours in females, brachytherapy or a combination of brachytherapy and EBRT is a viable alternative to surgical resection of the distal third of the urethra. Intraluminal brachytherapy is performed using a urinary Foleys catheter. Needle implantation is guided by ultrasonography and secured using the Syed-Neblett template. The CTV is defined based on urethra-cystoscopy and radiological findings, with an isotropic margin of 5 mm, on a contrast-enhanced planning CT scan. HDR or pulsed-dose-rate (PDR) brachytherapy is considered optimal from a radiobiological perspective, delivering a boost dose of 10–15 Gy following definitive EBRT or a definitive brachytherapy dose of 50–65 Gy. Reported adverse effects include mild to moderate cystitis, dysuria, pollakisuria, and gynaecological complications in female patients. Tumour extent is the only independent factor associated with local control, with no significant difference between EBRT and interstitial brachytherapy. Older studies report a 5-year local control rate of 64 % and a 7-year CSS of 49 % with a median radiation dose of 65 Gy. Combining brachytherapy with EBRT improves therapeutic outcomes and has shown to reduce local recurrence risk by a factor of 4.2, with the most pronounced benefit observed in patients with bulky tumours [4]

## Treatment for locally advanced disease

The significance of a multimodal treatment approach for managing locally advanced primary urethral carcinoma (T3-4N + M0) is consistently emphasized in the guidelines for improved tumour control and survival [1,23]. However, the sequencing of various modalities and the treatment paradigm remains inconsistent in practice and in literature. The following combination protocols are commonly recommended:

### Surgery followed by adjuvant radiotherapy

Organ-sparing surgery is often not feasible in advanced disease and generally a radical penectomy/urethrectomy, sometimes with a cystectomy or cysto-prostatectomy is necessary followed by adjuvant therapy to improve outcomes [23]. A retrospective analysis of 36 patients treated with surgery followed by radiotherapy has been reported from the conventional radiotherapy era, demonstrating a median survival time of 55.6 months, 5-years overall, and disease-free survival rate of 49 % and 23 %, respectively. The stratum of locally advanced cases demonstrated a 5-year OS of 33 % [27]. Better outcomes with the combination of surgery and RT as local therapy have been demonstrated in several other studies, with some of them proving the additional benefit of RT to the best survival possibilities [28]; especially in histology like Adenocarcinoma [29]. The recommendations by NCCN include radiotherapy to the post-operative bed and elective nodal basins (inguinal and pelvic lymph nodal groups) to a dose of 45–50.4 Gy, which is escalated to 60 Gy in case of positive margins or extra-nodal extension and an added boost to a total dose of 70 Gy is encouraged in cases of gross residual disease.

### Definitive radiotherapy with or without concurrent chemotherapy followed by salvage surgery

**Fig. 4:**
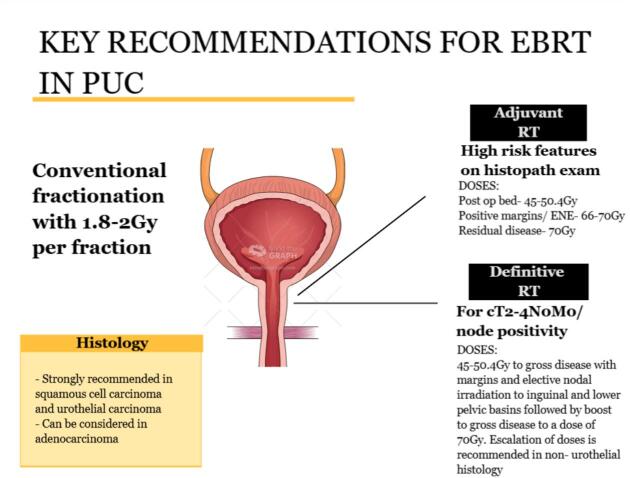
Key recommendations by NCCN for External Beam RT in PUC.

In patients with locally advanced PUC, definitive radiotherapy with or without concurrent chemotherapy has emerged as a viable organ-preserving alternative to radical surgery, particularly in patients unfit for or declining mutilating procedures. Various studies have demonstrated a beneficial effect on oncological outcomes, reporting overall survival rates of up to 83 % at 1 year following chemoradiation therapy [28,30]. The EAU and NCCN guidelines advocate for a multidisciplinary approach that includes radiotherapy as a key modality for tumour control in advanced stages [1,23]. Modern conformal techniques, such as intensity-modulated radiotherapy (IMRT) or VMAT with daily image guidance, enable dose escalation to 66–70 Gy to the gross disease and 45–50.4 Gy to elective nodal basins, thereby improving disease control while minimizing toxicity [23]. The NCCN 2024 guidelines emphasize the role of radiotherapy in the management of PUC, the key recommendations are depicted in Fig. 4. Concurrent chemotherapy, most commonly cisplatin, acts as a radiosensitizer and is associated with improved outcomes, particularly in urothelial histology [31]. Other regimens, such as 5-fluorouracil (5-FU) with mitomycin-C, have also demonstrated durable responses in selected series [[28], [32]]. In a study by Kent et al, 79 % of patients treated with definitive EBRT ± chemotherapy achieved complete response, with less than half requiring salvage surgery and a 5-years overall survival of 52 %. Gakis et al. and Dayyani et al. further report improved survival and recurrence-free outcomes with neoadjuvant chemotherapy in T3/T4 or node-positive disease [1], [30]. Organ preservation remains a central advantage, with several reports emphasizing genital preservation and acceptable toxicity profiles, particularly with contemporary conformal radiotherapy [[4], [22], [28]]. Salvage surgery is generally reserved for non-responders or cases of local recurrence.

## Treatment for metastatic or recurrent disease

Systemic chemotherapy with platinum-based regimes forms the cornerstone of management in these settings. Immunotherapy is an emerging option with PD-1/PD-L1 inhibitors (e.g. pembrolizumab, atezolizumab) for advanced or metastatic disease, particularly after chemotherapy failure, following the lines of management of bladder cancer [33], [34]. Palliative RT plays a vital role in symptomatic control at metastatic sites (e.g. bone pain, bleeding) while palliative urinary diversion procedures (e.g. suprapubic catheter) are reserved for symptom relief in obstructive cases.

### Follow-up and surveillance

Guidelines recommend regular monitoring with physical examination, cystoscopy, urine cytology and imaging (MRI, CT, or PET-CT) every 3–6 months for the first 2 years, then annually [1]. Long-term follow-up is tailored based on initial tumour stage, histology, and treatment received.

## Conclusion

Primary urethral cancers pose management challenges due to limited evidence, often presenting late in the elderly. Histology varies by gender, influencing treatment, which typically involves extensive surgery with adjuvant radiation and chemotherapy in advanced cases. In early stage disease, organ preservation with EBRT or brachytherapy is feasible, with surgery reserved for relapse. Contemporary series have demonstrated 5-year local control rates of 60–80 % with definitive EBRT or brachytherapy, which are comparable to those achieved with urethrectomy or cystoprostatectomy, but with substantially reduced morbidity. The integration of image guidance has significantly lowered rates of severe GU/GI toxicity compared with conventional techniques. While urethral stricture remains a notable late effect, its management is feasible with endoscopic or reconstructive interventions, and is often acceptable to patients given the preservation of sexual and urinary function. Refining treatment and establishing evidence-based guidelines will need better quality retrospective, or ideally prospective data, a challenge in this rare cancer.

## Patient consent

Verbal informed consent for the publication of this case report was obtained from the patient via telephone on 07.03.2025. The patient was informed about the nature and purpose of the publication, ensuring they understood that no personally identifiable information would be disclosed. The consent was documented in the patient’s medical records as per institutional guidelines.

## Authors’ contributions

All authors contributed to the conception, literature review, manuscript drafting and final approval of the version to be published.

## Ethics approval

This article is a narrative review and does not require ethical approval.

## Funding

This study did not receive any external funding.

## Declaration of competing interest

The authors declare that they have no known competing financial interests or personal relationships that could have appeared to influence the work reported in this paper.
